# Tumorigenesis promotes Mdm4-S overexpression

**DOI:** 10.18632/oncotarget.15552

**Published:** 2017-02-20

**Authors:** Vinod Pant, Connie A. Larsson, Neeraj Aryal, Shunbin Xiong, M. James You, Alfonso Quintas-Cardama, Guillermina Lozano

**Affiliations:** ^1^ Department of Genetics, M.D. Anderson Cancer Center, Houston, Texas, 77030, USA; ^2^ Department of Hematopathology, M.D. Anderson Cancer Center, Houston, Texas, 77030, USA; ^3^ Department of Leukemia, M.D. Anderson Cancer Center, Houston, Texas, 77030, USA

**Keywords:** splicing, Mdmx, Mdm4-S/Mdm4, CLL, transgenic mouse

## Abstract

Disruption of the *p53* tumor suppressor pathway is a primary cause of tumorigenesis. In addition to mutation of the *p53* gene itself, overexpression of major negative regulators of p53, MDM2 and MDM4, also act as drivers for tumor development. Recent studies suggest that expression of splice variants of *Mdm2* and *Mdm4* may be similarly involved in tumor development. In particular, multiple studies show that expression of a splice variant of *MDM4, MDM4-S* correlates with tumor aggressiveness and can be used as a prognostic marker in different tumor types. However, in the absence of prospective studies, it is not clear whether expression of *MDM4-S* in itself is oncogenic or is simply an outcome of tumorigenesis. Here we have examined the role of *Mdm4-S* in tumor development in a transgenic mouse model. Our results suggest that splicing of *Mdm4* does not promote tumor development and does not cooperate with other oncogenic insults to alter tumor latency or aggressiveness. We conclude that *Mdm4-S* overexpression is a consequence of splicing defects in tumor cells rather than a cause of tumor evolution.

## INTRODUCTION

Regulation of the p53 tumor suppressor and transcriptional activator is critical for normal cellular proliferation and survival. Mdm4, a homologue of Mdm2, is a *bona fide* negative regulator of p53 [1]. Genetic ablation of Mdm4 results in p53-dependent early embryonic lethality in mice [2-4], while deletion of Mdm4 in adult mouse tissues leads to subtle activation of p53 that can be potentially harnessed for therapeutic purposes [5]. Mdm4 inhibits the transcriptional activity of p53 by binding to and masking its transcriptional activation domain. In addition, Mdm4 heterodimerizes with Mdm2 through its RING domain to generate an effective E3-ubiquitin ligase that degrades p53 protein during early embryogenesis [6, 7].

Overexpression and amplification of *MDM4* is a common theme associated with suppression of the p53 pathway in a range of human tumors [8-13]. In addition, multiple studies have highlighted the expression of *MDM4* spliced variants in different tumors types [11, 14]. One *MDM4* spliced variant, *MDM4-S* that skips exon 6 and prematurely terminates in exon 7 has been the subject of close scrutiny in recent years [15]. The *MDM4-S* transcript potentially encodes a truncated Mdm4 protein carrying only the N-terminal p53-binding domain along with 13 novel amino acids [16]. Overexpression of *MDM4-S* has been linked to poor prognosis in osteosarcoma, soft tissue sarcoma, breast cancer, glioblastoma, melanoma, and chronic lymphocytic leukemia [17-21]. MDM4-S lacks an internal autoinhibitory sequence [22] and previous overexpression studies indicated that nuclear localized MDM4-S acts as a potent inhibitor of p53 activity and thus likely functions as an oncogene [15, 16]. Recent studies however suggest that the *MDM4-S* transcript is susceptible to nonsense mediated decay and thus acts as a critical determinant of MDM4 expression in tumors with mutant p53 [17, 18]. It is not clear why tumor cells with a defective p53 pathway would require a reduction in full-length Mdm4 levels.

While the oncogenic potential of the full length Mdm4 protein has been clearly demonstrated by mouse studies [23], similar studies characterizing the Mdm4-S form are lacking. In the absence of prospective animal studies, it is not clear whether *Mdm4-S* overexpression is a cause or a consequence of tumorigenesis. To address this issue, a knock-in mouse was recently generated [24]. Unfortunately, the mouse was embryonic lethal due to excessive p53 activity. This suggests that overexpression of Mdm4-S form is not sufficient to overcome the loss of endogenous full length Mdm4. Alternatively, it is possible that loss of the C-terminus RING domain function in these mice lead to pre-natal lethality [6, 7].

We recently examined *MDM4-S* expression in B-cell chronic lymphocytic leukemia (B-CLL, a B-cell associated malignancy) patient samples. Interestingly, over 47% of patient samples overexpressed *MDM4-S* mRNA compared to normal healthy volunteer controls. To gain more insight into the role of *MDM4-S* splice variant in B-cell malignancies, we generated a transgenic mouse wherein Mdm4-S overexpression was regulated by the Ig gene VH promoter along with the intronic enhancer and restricted to the B-cell lineage. This strategy prevented the early embryonic lethality of truncated Mdm4 expression and allowed us to investigate the significance of Mdm4-S overexpression in development of B-cell hematopoietic malignancies in mice. Herein, we present the results obtained with this mouse model and provide evidence that splicing of *Mdm4* is a consequence rather than a cause of tumorigenesis.

## RESULTS

### *MDM4-S* variant is overexpressed in B-CLL patient samples

**Figure 1:**
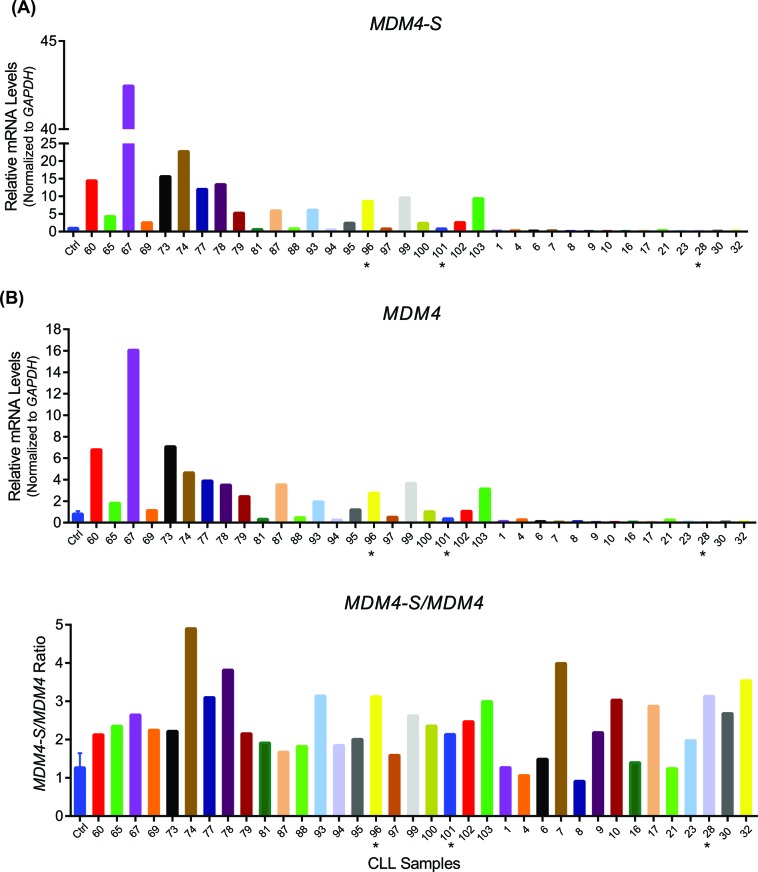
***Mdm4-S*** is overexpressed in B-CLL. **A**. Real-time PCR showing *MDM4-S* (upper panel) and *MDM4* (lower panel) expression in B-CLL patient samples. **B**. *MDM4-S/MDM4* ratio in B-CLL patient samples. Ctrl is the normal lymphocyte RNA control. Samples were normalized to Ctrl that was set to 1. * indicates samples with 17p deletion which spans the p53 locus.

Recent studies have linked *MDM4-S* overexpression to poor prognosis in human cancer [17]. In order to test whether the *MDM4-S* form is overexpressed in B-CLL, we carried out an initial screening of 36 unselected samples obtained from patients with newly diagnosed B-CLL prior to undergoing standard chemoimmunotherapy with fludarabine, cyclophosphamide, and rituximab (i.e. FCR regimen) at the MD Anderson Cancer Center (Table 1). Overall, 47% (17/36) of patient samples showed increased expression of the *MDM4* splice variant, *MDM4-S* to varying extents ranging from 2 fold to 42 fold as compared with B-cells obtained from normal healthy volunteers (Figure 1A). Next, we compared the expression of full length *MDM4* and the corresponding *MDM4-S/MDM4* ratio in these patient samples. The *MDM4-S/MDM4* ratio in these patients was also noticeably higher than normal controls (Figure 1B). Of note, during the conduction of this project a separate study from China also reported higher levels of *MDM4-S* in a larger cohort of B-CLL patients correlating with poor prognosis after FCR therapy [20]. Overall, these two independent reports clearly implicate *MDM4-S* overexpression in the pathogenesis of B-CLL.

**Table 1:** Clinical and biological characteristics of chronic lymphocytic leukemia patients

CLL No.	Age yrs	Sex M/F	Rai stage	B2M μg/ml	HGB g/dL	PLT x10^9^/L	WBC x10^9^/L	Absolute Lympho /μl	% ATM deletion (FISH)	% Deletion 13q (FISH)	% Trisomy 12 (FISH)	% p53 deletion (FISH)	IgVH mutational status	ZAP70	Mdm2 SNP309 rs2279744	Mdm4-S >2-fold
1	61	M	1	3.3	11.7	205	119.2	113240	89	0	0	0	UNMUTATED	+	TT	No
4	67	M	3	4	9.5	168	177.6	166944	ND	ND	ND	ND	UNMUTATED	+	GT	No
6	61	M	4	4.2	16.5	50	161.6	145440	0	0	46	0	MUTATED	+	ND	No
7	50	M	4	5.3	13.6	97	45.1	39237	61	0	0	0	UNMUTATED	+	TT	No
8	55	M	2	3.2	14.6	236	63.4	53256	0	65.5	0	0	MUTATED	-	GT	No
9	57	M	1	4.5	12.8	180	135.2	123032	0	78	0	0	UNMUTATED	+	GT	No
10	59	M	3	2.7	9.8	191	137.6	125216	0	0	62.5	0	UNMUTATED	+	GT	No
16	53	M	4	5.8	12.9	79	32.6	29666	0	0	0	0	UNMUTATED	-	GG	No
17	66	M	1	6.2	12.4	116	151.4	140802	94	96.5	0	0	UNMUTATED	-	TT	No
21	66	M	3	3	10.9	116	252.5	242400	0	95.5	0	0	NR	+	GT	No
23	61	F	2	2.3	13.5	266	164.9	158304	95.5	86.5	0	0	UNMUTATED	-	GT	No
28	65	M	2	4	12.4	116	158.5	142650	0	69	0	78	MUTATED	ND	TT	No
30	62	F	0	2.6	12.2	177	153.7	144478	0	92.5	0	0	NR	-	TT	No
32	58	F	1	2.4	11.2	101	139.2	139200	0	71	0	0	MUTATED	-	GG	Yes
60	62	F	2	6.9	11.4	225	114.3	102870	0	80.5	0	0	MUTATED	+	GG	Yes
65	55	F	1	3	11.9	159	222.8	207204	0	0	0	0	UNMUTATED	-	TT	Yes
67	54	F	2	2.6	15.9	201	52.2	48024	0	40	0	0	UNMUTATED	-	GT	Yes
69	63	M	4	3.4	9.6	56	5.9	5428	10	0	0	0	UNMUTATED	+	GT	Yes
73	51	F	2	1.7	13.7	140	36.3	33033	0	80	0	0	MUTATED	-	TT	Yes
74	74	M	4	3.3	13.4	88	161.8	148856	96	95	0	0	UNMUTATED	+	GT	Yes
77	60	F	4	3.2	9.9	80	120	115200	0	73.5	0	0	MUTATED	-	GT	Yes
78	73	F	2	3.1	11.4	192	177.9	167226	0	0	73	0	UNMUTATED	+	TT	Yes
79	64	M	1	4.5	11.5	168	100.4	90360	21.5	52.5	0	0	UNMUTATED	+	TT	Yes
81	62	M	3	3.1	10.6	254	74.2	69748	0	0	57.5	0	UNMUTATED	+	GT	No
87	57	F	2		12.1	129	67.1	55693	0	0	53	0	MUTATED	ND	TT	Yes
88	63	F	2	6	11.9	201	267.9	235752	ND	ND	ND	ND	UNMUTATED	+	TT	No
93	44	M	4	4.2	8.9	57	31	30070	0	80.5	0	0	MUTATED	-	GT	Yes
94	53	F	3	3.1	10.4	163	196.4	182652	0	0	0	0	UNMUTATED	+	GT	No
95	50	F	1	2	12.4	193	121.3	115235	0	89	0	0	MUTATED	ND	GT	Yes
96	58	F	2	4	12.5	220	221.4	210330	0	62.5	0	88.5	UNMUTATED	-	TT	Yes
97	58	M	4		9.5	15	71.5	71500	94.5	0	0	0	UNMUTATED	+	TT	No
99	58	F	3	11.4	10	131	120.5	110860	23	12	0	0	UNMUTATED	+	TT	Yes
100	62	M	3	3.5	10.2	111	141	138180	0	0	0	0	MUTATED	-	GT	Yes
101	49	M	2	2.8	13.2	119	94.5	91665	0	92.5	0	65	UNMUTATED	+	GG	No
102	60	F	2	4.9	12.6	195	84.5	47320	0	0	0	0	UNMUTATED	+	GT	Yes
103	71	M	3	6	8.9	190	182.6	173470	55.5	0	82	0	UNMUTATED	+	TT	Yes

Abbreviations: F: female, M: male, B2M: beta 2 microglobulin, HGB: hemoglobin, PLT: platelet count, WBC: white blood cell count, absolute lympho: absolute lymphocyte count, FISH: fluorescence in situ hybridization, ZAP70: Zeta Chain of T Cell Receptor Associated Protein Kinase 70kDa, IgVH: heavy chain of the immunoglobulin gene, +: positive, – : negative, ND: not determined.

### Generation of Mdm4-S overexpression mouse

**Figure 2:**
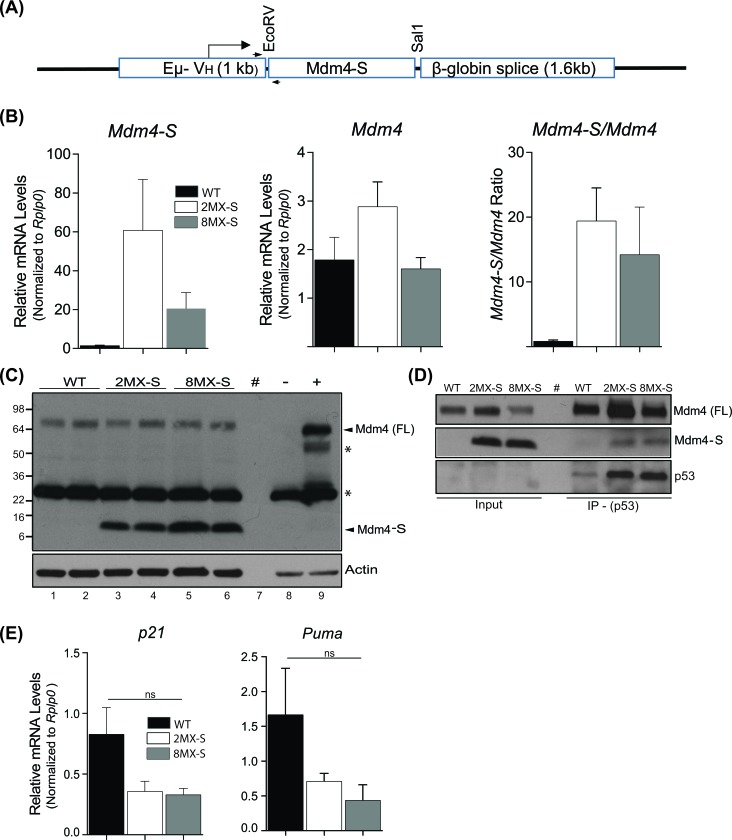
Generation and characterization of ***Mdm4-S*** transgenic mouse. **A**. Design of transgenic mouse Mdm4-S construct. **B**. Real-time PCR showing *Mdm4-S* and *Mdm4* mRNA expression, and the *Mdm4-S/Mdm4* ratio in transgenic mouse spleens. *n* = 3, ±SEM. **C**. WB of protein lysates from transgenic mouse spleens showing Mdm4-S and Mdm4 expression. - and + represent negative and positive controls for Mdm4 expression respectively **D**. Co-IP of Mdm4-S with an anti-p53 antibody. **E**. RT-qPCR for p53 targets in mouse spleens. *n* = 3, ±SEM. WT: wild type, WB: western blot, IP: immunoprecipitation, #: blank lane, *: non specific, ns: not significant.

In order to examine a possible pathogenetic role of *MDM4-S* overexpression in B-CLL, we cloned a murine *Mdm4-S* cDNA into the pBSVE6BK vector (Figure 2A). This transgene vector harbors the Ig gene VH promoter and the intronic enhancer and has been shown to drive gene expression restricted to the B-cell lineage, thus allowing us to specifically target Mdm4-S expression in the cell of origin of B-CLL [25]. We confirmed the presence of the *Mdm4-S* transgene in tail snip DNA of 4 of 10 founder mice (data not shown). Based on subsequent characterization, we pursued two lines, henceforth called *2MX-S* and *8MX-S*, for germline transmission and follow-up studies.

### Characterization of Mdm4-S overexpression mouse lines

As our original strategy targeted overexpression of *Mdm4-S* mRNA in the B-cell lineage, we first examined *Mdm4-S* expression in the spleen (a predominantly B-cell organ) of these mice. Compared to wild type controls, *2MX-S* and *8MX-S* mouse spleens showed 60- and 20-fold higher expression of *Mdm4-S* mRNA, respectively (Figure 2B). Next, we analyzed the mRNA expression of full length *Mdm4* and the corresponding *Mdm4-S/Mdm4* ratio in these two mouse lines. The *Mdm4-S/Mdm4* ratio was significantly higher (19x and 13x, respectively) in these mice compared to the wild type controls. Further, we examined the Mdm4-S protein levels in the spleens of these mice. An increased abundance of Mdm4-S protein was detected in the spleens of *2MX-S* and *8MX-S* mice, while no difference in Mdm4 levels was observed across the different mouse genotypes (Figure 2C). Of note, no Mdm4-S protein expression was noted in wild type mouse spleens indicating lack of detectable levels of Mdm4-S in normal animal tissues.

Mdm4-S encodes a truncated Mdm4 protein with only the N-terminal p53 binding domain intact [15]. To confirm whether the overexpressed Mdm4-S protein in our transgenic mice similarly binds to endogenous p53, we performed co-immunoprecipitation experiments. We immunoprecipitated protein lysates from spleens of *wild type*, *2MX-S* and *8MX-S* mice with an anti-p53 antibody and subjected it to immunoblotting with anti-Mdm4 antibody (Figure 2D). As expected, we could pull down Mdm4-S from 2MX-S and 8MX-S protein lysate with a p53 antibody confirming the interaction between the two proteins, though the interaction appeared much weaker compared to Mdm4 and p53 proteins. We also examined p53 transcriptional activity in the spleens of these transgenic mice (Figure 2E). Interestingly, mRNA expression of p53 downstream targets *p21* and *Puma* was slightly lower in *2MX-S* and *8MX-S* mouse spleens compared to their wild type counterpart, but the difference was not statistically significant.

**Figure 3:**
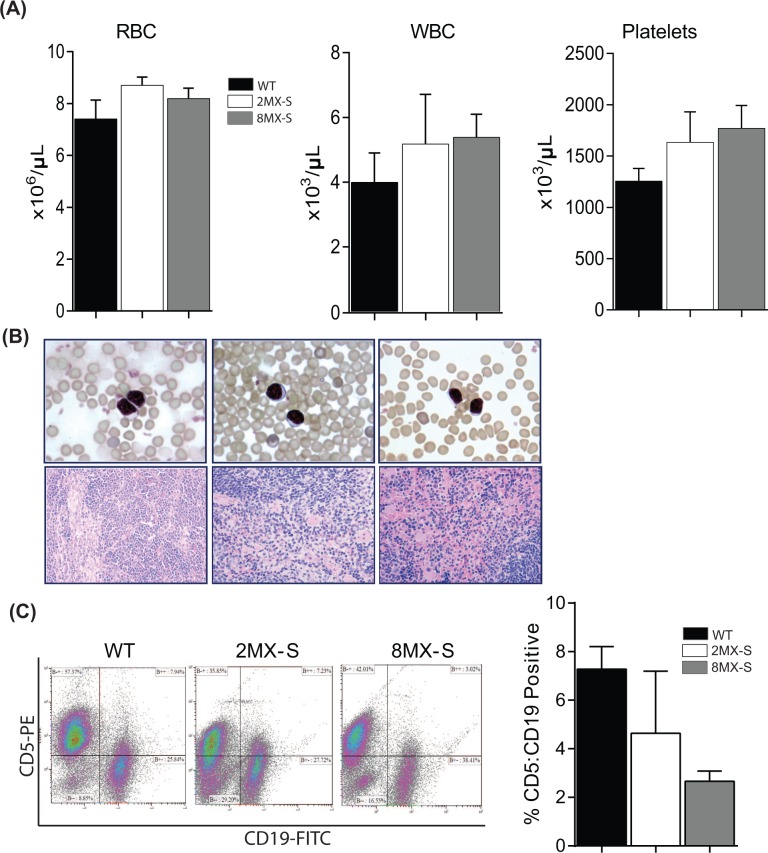
Overexpression of ***Mdm4-S*** does not promote B-cell associated malignancy in mice. **A**. Complete CBC analysis of WT (*n* = 4), 2MX-S (*n* = 7) and 8MX-S (*n* = 11) transgenic mice. ±SEM. **B**. Top panel- Representative peripheral blood smear slides from WT, 2MX-S and 8MX-S mice. 100x magnification. Bottom panel- Representative Hematoxylin and eosin stained sections of spleens from WT, 2MX-S and 8MX-S mice. 40x magnification. **C**. Flow cytometry analysis for CD5 and CD19 double positive cells in splenocytes generated from WT, 2MX-S and 8MX-S mice. *n* = 3, ±SEM. WT: wild type.

In order to determine whether overexpression of the Mdm4-S variant in B-cells leads to hematological alterations in transgenic mice, we next examined hemograms of *2MX-S* and *8MX-S* mice (Figure 3A). No variation in complete blood cell count was observed in 16-20 month old *2MX-S* and *8MX-S* mice when compared to age matched wild-type control mice. In addition, blood smears from the transgenic mice of different ages also failed to reveal any significant morphological or quantitative differences compared to smears obtained from normal controls. No sign of malignant transformation was detected (Figure 3B). Next, we proceeded with complete histo-pathological examination of spleens obtained from *2MX-S* and *8MX-S* mice of different ages. Again, no splenomegaly or other obvious signs of disease were noted (Figure 3B). Finally, to evaluate whether overexpression of the Mdm4-S variant leads to a B-cell malignant phenotype in mice, we performed flow cytometry to evaluate CD5 and CD19 co-expression on splenocytes. CD5:CD19 double positivity is a characteristic phenotypic feature of B-CLL cells [26]. Remarkably, no differences in double positive cell counts was observed in wild type and transgenic mouse spleens (Figure 3C). Of note, for some unknown reasons the percentage of double positive CD5:CD19 cells in *8MX-S* mice was noticeably lower than the wild type controls.

### Tumorigenic profile and survival of Mdm4-S overexpressing transgenic mouse lines

**Figure 4:**
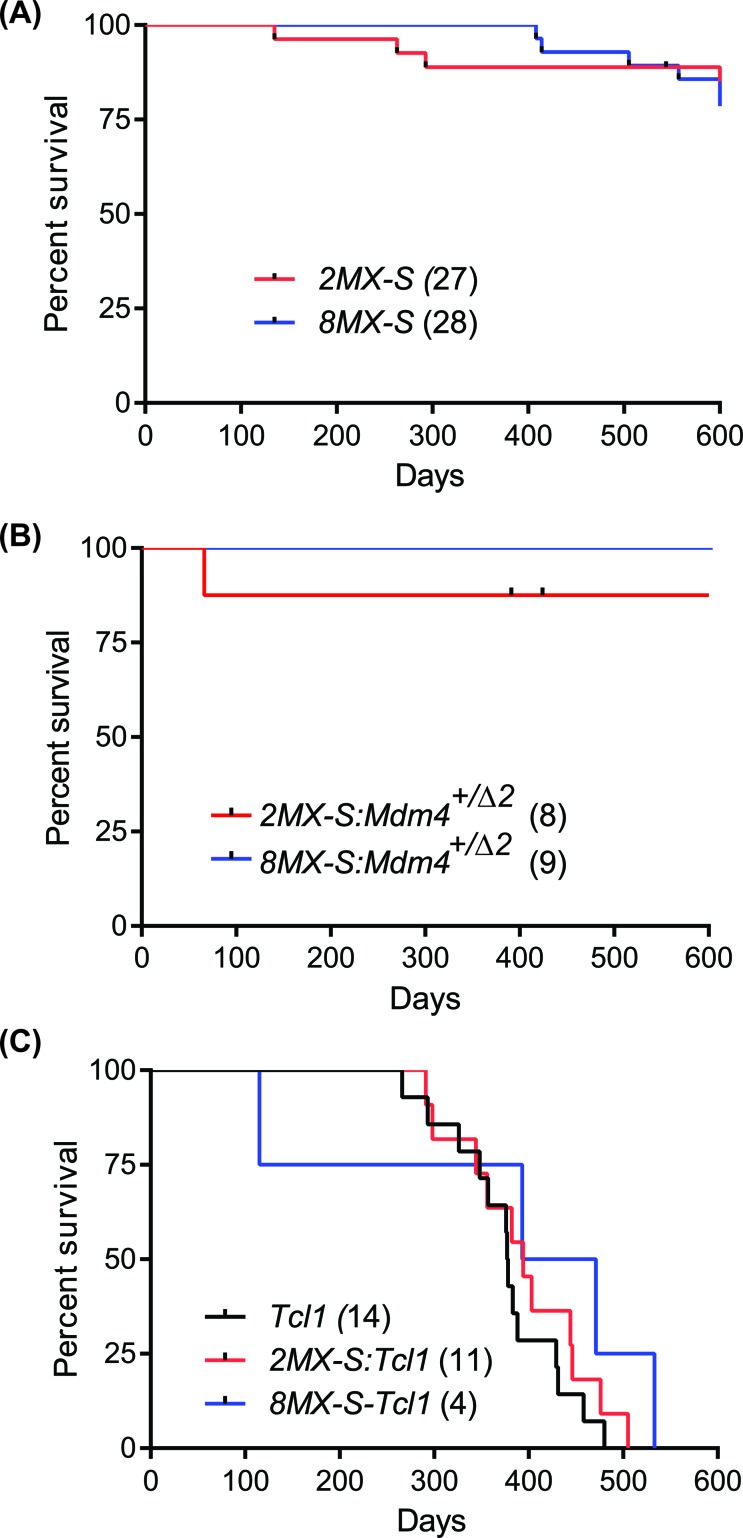
Mdm4-S overexpression does not cooperate with other oncogenic insults **A**. Kaplan-Meier survival curve of 2MX-S and 8MX-S transgenic mouse lines. **B**. Kaplan-Meier survival curve of *2MX-S:Mdm4^+/Δ2^* and 8MX-S:*Mdm4^+/Δ2^* mouse lines. **C**. Kaplan-Meier survival curve of *TCL1*, *2MX-S:TCL1* and *8MX-S:TCL1* mice.

To evaluate the physiological impact of Mdm4-S overexpression in mice of both transgenic lines, we generated cohorts of *2MX-S* and *8MX-S* mice and monitored them for tumor development and overall survival (Figure 4A). Surprisingly, none of the transgenic mice overexpressing Mdm4-S developed a B-cell malignant phenotype. In addition, no apparent change in overall survival was noted in Mdm4-S overexpressing mouse lines. Most mice were sacrificed at the end of study (i.e. 600 days).

Recent studies have reported that increased splicing at the *MDM4* locus counters the expression of full length Mdm4 [17, 18]. In addition, an increase in *MDM4-S/MDM4* ratio has been proposed as a marker of poor prognosis in different human cancers [17]. In order to test whether an increase in *Mdm4-S* in conjunction with a corresponding decrease in full length *Mdm4* promotes B-cell malignancy, we crossed Mdm4-S transgenic mice to *Mdm4^Δ2^* mice. *Mdm4^Δ2^* is a null allele previously generated in our lab [27]. We monitored a cohort of *2MX-S:Mdm4^+/Δ2^* and *8MX-S: Mdm4^+/Δ2^* mice for tumor development. Interestingly, even with genetically half gene dosage of the full length *Mdm4*, *2MX-S:Mdm4^+/Δ2^* and *8MX-S:Mdm4^+/Δ2^* mice did not develop any B-cell malignant phenotype and lived a normal life span (Figure 4B).

Lastly, we tested whether *Mdm4-S* overexpression cooperates with other oncogenes in tumorigenesis. To that end, we crossed *Mdm4-S* mice with *Eμ-TCL1* transgenic mice, a prototypical B-CLL mouse model expressing the T cell leukemia-1(*TCL1*) transgene in B-cells that displays similar clinical and therapeutic response properties to human B-CLL [28]. We generated a cohort of *2MX-S*:*TCL1*, and *8MX-S*:*TCL1* mice and investigated whether cooperation of these two genetic events leads to earlier tumorigenesis and shorter survival in these compound mice (Figure 4C). Intriguingly, genetic combination of these two events did not alter the B-CLL latency or the overall survival of these mice. Failure to exacerbate the disease profile ruled out cooperativity between these two oncogenic insults.

### Mdm4-S overexpression is a consequence of tumorigenesis

**Figure 5:**
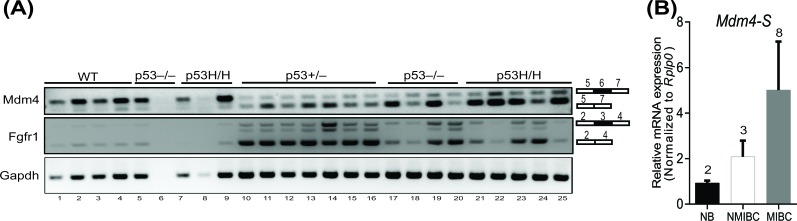
Aberrant gene splicing is a consequence of tumorigenesis **A**. RT-PCR analysis to show *Mdm4-S* and *Fgfr1* splice variant expression in normal spleens (lane 1-9) and tumors (lane 10-25) from *wild type*, *p53^+/ −^*, *p53 ^−/−^* and *p53^R172H/H^* mice. *Gapdh* is used as a control. **B**. Real time PCR analysis for *Mdm4-S* expression in mouse normal bladder (NB), non-muscle invasive bladder cancer (NMIBC) and muscle invasive bladder cancer (MIBC) samples.

Finally, given the lack of an overt malignant phenotype, we investigated whether overexpression of *Mdm4-S* is a consequence rather than a cause of tumorigenic events. To that end, we isolated spleens from 3 week old *p53^+/+^*, p53^−/−^, and *p53^R172H/H^* (inheriting 2 copies of the p53R172H hotspot mutation) pre-tumorigenic mice. Separately, we also collected different types of tumors (lymphoma and osteosarcoma) that originated in *p53 ^+ / −^*, *p53 ^−/−^*, and *p53^R172H/H^* mice. We isolated RNA from these normal pre-tumorigenic spleens and mouse tumors and performed RT-PCR analysis to compare the *Mdm4-S* expression in these biological samples (Figure 5A). Notably, we did not observe high levels of *Mdm4-S* mRNA in the spleens of pre-tumor mice of any genotype (Figure 5A, lanes 1-9). However, the *Mdm4-S* mRNA was prominently expressed in all 16 mouse tumor samples examined (Figure 5A, lanes 10-25). Next, we tested whether splicing of other genes is also altered in the above mouse spleens and tumor samples. To that end we examined the expression of *Fibroblast Growth Factor Receptor 1* (*Fgfr1*) mRNA in these biological samples. *Fgfr1* produces a splice variant by exon 3 skipping in tumors. Similar to the *Mdm4-S* expression, very little expression of *Fgfr1* splice variant was observed in pre-tumorigenic spleens (Figure 5). In contrast, a noticeable expression of *Fgfr1* splice variant was evident in all the murine tumor samples.

Finally, to rule out the possibility that pre-existing genomic instability in the *p53^+/−^*, *p53^−/−^*, and *p53^R172H/H^* mice promoted splicing events in the above experiment, we also screened for *Mdm4-S* expression in non-muscle invasive and muscle invasive bladder cancer (NMIBC and MIBC) samples that originated in wild type mice after treatment with N-Butyl-N-(4-hydroxybutyl)nitrosamine (*BBN*), a urothelial carcinogen. Again, real time PCR analysis indicated that increased *Mdm4-S* expression was confined to tumor samples (Figure 5B). Relatively higher *Mdm4-S* mRNA levels were noted in MIBC samples compared to NMIBC samples, though this was not statistically significant. Altogether, these results highlight that expression of gene splice variants is a phenomenon associated with tumorigenesis but not necessarily the cause of it.

## DISCUSSION

Disruption of the p53 pathway by overexpression of MDM4 is a common theme in many different types of human cancers. Recent studies have shown that the *MDM4* locus produces multiple splice variants in tumors as well as in culture cells that are exposed to stress conditions [15]. In particular, one such splice variant *MDM4-S*, generated by exon 6 skipping correlates with poor prognosis in different types of cancers [11, 17, 20]. However, in the absence of prospective studies, it is not clear whether *MDM4-S* overexpression is a cause or consequence of tumorigenesis.

Following our initial observations that *MDM4-S* transcript is overexpressed in B-CLL and to directly address this question, we generated a transgenic mouse in which *Mdm4-S* overexpression was restricted to the B-cell lineage. This strategy allowed us to avoid the embryonic death fate and directly address the significance of *Mdm4-S* overexpression in B-cell associated tumorigenesis. Notably, we validated *Mdm4-S* expression in spleens of the transgenic mice and also confirmed the interaction between transgene derived Mdm4-S and the endogenous p53 protein. A relatively weak p53-Mdm4-S interaction along with slight decrease in basal p53 transcriptional activity was observed. Nonetheless, overexpression of *Mdm4-S* variant in the B-cell lineage did not result in a malignant phenotype thereby ruling out untoward suppression of the p53 pathway.

Recent studies indicate that *MDM4-S* transcripts regulate the abundance of MDM4 protein and that the *MDM4-S/MDM4* ratio correlates with tumor aggressiveness [17]. In order to test this we modulated the *Mdm4-S/Mdm4* ratio by crossing *Mdm4-S* transgenic mice with *Mdm4^Δ2^* mice. Surprisingly, decreasing the levels of full-length Mdm4 by 50% and thereby increasing the *Mdm4-S/Mdm4* ratio also failed to promote B-cell linked tumorigenesis. These results are similar to the previous report that showed decrease in *Mdm4-S/Mdm4* ratio had little effect on adult mouse tissues [24]. Furthermore, genetic crosses with a prototypical B-CLL mouse model also showed that Mdm4-S expression does not cooperate with other oncogenic insults such as *TCL1* transgene expression to alter B-cell tumor latency in mice. Altogether, these results argue against the speculated role of *Mdm4-S* expression as an oncogene in B-cell lymphomagenesis.

Finally, it has been proposed that *MDM4-S* overexpression can serve as an effective biomarker for p53 pathway attenuation in cancers than p53 gene mutation itself [17]. This was clearly correlative in osteosarcoma and breast cancers [17, 21]. However, we did not observe any correlation between *MDM4-S* expression and p53 mutation status in the human B-CLL samples (Figure 1). Of note, we did not analyze expression levels of Mdm2 or other p53 inhibitors and therefore cannot rule out p53 pathway attenuation by other mechanisms. Importantly, prominent *Mdm4-S* expression was restricted to mouse tumors with genetic loss of p53 function and no expression was observed in normal mouse spleens (Figure 5). Moreover, Mdm4-S protein was not detected in mouse tumors that exhibited high levels of *Mdm4-S* mRNA (data not shown). This is in agreement with the recent reports that suggest that the endogenous *Mdm4-S* transcript is susceptible to nonsense mediated decay [18, 24, 29].

Nonetheless, the expression of splice variant of *Mdm4* and one other gene was clearly evident in a range of mouse tumor samples. Previously, genome-wide studies have revealed that large scale alterations in alternative splicing are associated with tumorigenesis [30, 31] and mutations in *SF3B1*, a core component of spliceosome machinery correlate with disease aggressiveness and shorter survival in CLL [32]. Furthermore, it has been proposed that *MDM4* alternative splicing serves as a key sensor of defects in the constitutive spliceosomal machinery that result in activation of the p53 response [29]. In keeping with this function, it is no surprise that 1). *p53* is commonly lost or mutated due to selective pressure in tumors with expression of *MDM4-S* and 2). *MDM4-S* expression correlates with tumor aggressiveness and poor prognosis in different human cancers [17]. The relevance of our study is that we have demonstrated that overexpression of *Mdm4-S* in B-cells is not oncogenic *per se* and is rather a consequence of tumorigenesis, at least in CLL. Thus, our *in vivo* study supports the idea of using *Mdm4-S* mRNA levels as a possible biomarker but cautions against over-interpreting splicing data for therapeutic purposes.

## MATERIALS AND METHODS

### Human B-CLL patient sample collection

Human samples were obtained from patients with newly diagnosed B-CLL prior to receiving standard frontline therapy with the fludarabine, cyclophosphamide, and rituximab (i.e. FCR regimen). Samples were obtained from the Department of Leukemia Tissue Bank at MD Anderson Cancer Center where they had been stored after being acquired during routine diagnostic assessments in accordance with the regulations and protocols approved by the MD Anderson Cancer Center Investigational Review Board. Signed informed consent was obtained from all patients and research was conducted in accordance with the Declaration of Helsinki. Samples were examined by a hematopathologist and graded per Rai stage. ZAP70 expression and IgVH mutation status as prognostic markers was confirmed. Chromosome 12 trisomy, 13q deletion, ATM deletion and p53 deletion were assessed by fluorescence *in situ* hybridization (FISH) analysis. Mdm2 SNP309 status was also determined.

### Generation of transgenic *Mdm4-S* mice

Mouse *Mdm4-S* cDNA was cloned as an EcoRV-Sal1 fragment into the pBSVE6BK vector. Linearized vector was injected into blastocysts to generate chimeric mice by the Genetically Engineered Mouse Facility at the MD Anderson Cancer Center. DNA from mouse tail snips was PCR amplified with vector specific forward primer (acccagatgtcccttcttctccag) and Mdm4-S specific reverse primer (gactcgagtcagttctttttctgggattg) to confirm the presence of the transgene. All mouse studies were approved by MD Anderson Cancer Center IACUC. *TCL1* mice were a gift from Dr. Croce (Ohio State University, Columbus, OH). *Mdm4^Δ2^* mice were previously generated in our lab [27].

### RNA isolation and real time PCR

RNA was isolated from mouse spleen or human B-CLL patient lymphocytes using TRIzol (Thermo Fisher Scientific). After DNase1 treatment and phenol-chloroform extraction, RNA was precipitated and suspended in DNase/RNase free water. One microgram of RNA was used for first strand synthesis (First-Strand cDNA Synthesis Kit, GE Life Sciences). Real time PCR was carried out as previously described [6]. Real time PCR conditions and primers for amplifying *MDM4-S*, *MDM4* and *GAPDH* from human B-CLL samples were originally described by Bartel et al [11]. Primers used for RT-qPCR for amplifying *Mdm4-S* (for-tgtgaaagatccaagccctct, rev-tgttgcaccgtgctgtgtta), *Mdm4* (for-ggaaaagcccaggtttgacc, rev-gccaaatccaaaaatcccact) and *Rplp0* (for-ccctgaagtgctcgacatca, rev-tgcggacaccctccagaa).

Primers used for semi-quantitative RT-PCR for amplifying mouse- *Mdm4-S* (for-tgtggtggagatcttttggg and rev-tcagttctttttctgggattgg), *Fgfr1* (for-gccttgttaccaacctctaac and rev-gaaccttgtagcctccaattc) and *Gapdh* (for-aggttgtctcctgcgacttca and rev-ggtggtccagggtttcttactc).

### Western blotting and immunoprecipitation

Mouse spleen were lysed in NP-40 buffer and 100μg of protein lysate was resolved on 4-15% gradient gels (BioRad) and Immunoblotted with either anti-p53 (CM5, Vector Biolabs, 1:1000) or anti-Mdm4 (MX-82, Calbiochem, 1:500) or anti-actin (AC15, Sigma, 1:5000) antibodies. For IP, 1 mg of protein lysate was pulled down with anti-p53 antibody (CM5) and immunoblotted with either Mdm4 antibody (MX-82, Calbiochem, 1:500) or p53 antibody (FL393, Santa Cruz biotechnology, 1:500). 10% of protein lysate was used as input.

### CBC analysis and flow cytometry

Blood collected from heart puncture was analyzed by Dept. of Veterinary Medicine hematology core at MD Anderson Cancer Center. Blood smear slides were stained with Hema-3 fixative (Fisher Healthcare) and microscopically analyzed by a hematopathologist. Splenocytes isolated from wild type, 2MX-S and 8MX-S mouse spleens were treated with RBC lysis buffer and labelled with CD19-FITC (BD Pharmingen) and CD5-PE (eBiosciences) antibodies in binding buffer. Flow analysis was carried out by flow cytometry core at MD Anderson Cancer Center.

## References

[1] Shvarts A, Steegenga WT, Riteco N, van Laar T, Dekker P, Bazuine M, van Ham RC, van der Houven van Oordt W, Hateboer G, van der Eb AJ, Jochemsen AG (1996). MDMX: a novel p53-binding protein with some functional properties of MDM2. EMBO J.

[2] Parant J, Chavez-Reyes A, Little NA, Yan W, Reinke V, Jochemsen AG, Lozano G (2001). Rescue of embryonic lethality in Mdm4-null mice by loss of Trp53 suggests a nonoverlapping pathway with MDM2 to regulate p53. Nat Genet.

[3] Migliorini D, Lazzerini Denchi E, Danovi D, Jochemsen A, Capillo M, Gobbi A, Helin K, Pelicci PG, Marine JC (2002). Mdm4 (Mdmx) regulates p53-induced growth arrest and neuronal cell death during early embryonic mouse development. Mol Cell Biol.

[4] Finch RA, Donoviel DB, Potter D, Shi M, Fan A, Freed DD, Wang CY, Zambrowicz BP, Ramirez-Solis R, Sands AT, Zhang N (2002). mdmx is a negative regulator of p53 activity in vivo. Cancer Res.

[5] Garcia D, Warr MR, Martins CP, Brown Swigart L, Passegue E, Evan GI (2011). Validation of MdmX as a therapeutic target for reactivating p53 in tumors. Genes Dev.

[6] Pant V, Xiong S, Iwakuma T, Quintas-Cardama A, Lozano G (2011). Heterodimerization of Mdm2 and Mdm4 is critical for regulating p53 activity during embryogenesis but dispensable for p53 and Mdm2 stability. Proc Natl Acad Sci U S A.

[7] Huang L, Yan Z, Liao X, Li Y, Yang J, Wang ZG, Zuo Y, Kawai H, Shadfan M, Ganapathy S, Yuan ZM (2011). The p53 inhibitors MDM2/MDMX complex is required for control of p53 activity in vivo. Proc Natl Acad Sci U S A.

[8] Riemenschneider MJ, Knobbe CB, Reifenberger G (2003). Refined mapping of 1q32 amplicons in malignant gliomas confirms MDM4 as the main amplification target. International journal of cancer.

[9] Danovi D, Meulmeester E, Pasini D, Migliorini D, Capra M, Frenk R, de Graaf P, Francoz S, Gasparini P, Gobbi A, Helin K, Pelicci PG, Jochemsen AG (2004). Amplification of Mdmx (or Mdm4) directly contributes to tumor formation by inhibiting p53 tumor suppressor activity. Mol Cell Biol.

[10] Ramos YF, Stad R, Attema J, Peltenburg LT, van der Eb AJ, Jochemsen AG (2001). Aberrant expression of HDMX proteins in tumor cells correlates with wild-type p53. Cancer Res.

[11] Bartel F, Schulz J, Bohnke A, Blumke K, Kappler M, Bache M, Schmidt H, Wurl P, Taubert H, Hauptmann S (2005). Significance of HDMX-S (or MDM4) mRNA splice variant overexpression and HDMX gene amplification on primary soft tissue sarcoma prognosis. International journal of cancer.

[12] Laurie NA, Donovan SL, Shih CS, Zhang J, Mills N, Fuller C, Teunisse A, Lam S, Ramos Y, Mohan A, Johnson D, Wilson M, Rodriguez-Galindo C (2006). Inactivation of the p53 pathway in retinoblastoma. Nature.

[13] Wasylishen AR, Lozano G (2016). Attenuating the p53 Pathway in Human Cancers: Many Means to the Same End. Cold Spring Harbor perspectives in medicine.

[14] Mancini F, Di Conza G, Moretti F (2009). MDM4 (MDMX) and its Transcript Variants. Current genomics.

[15] Rallapalli R, Strachan G, Cho B, Mercer WE, Hall DJ (1999). A novel MDMX transcript expressed in a variety of transformed cell lines encodes a truncated protein with potent p53 repressive activity. J Biol Chem.

[16] Rallapalli R, Strachan G, Tuan RS, Hall DJ (2003). Identification of a domain within MDMX-S that is responsible for its high affinity interaction with p53 and high-level expression in mammalian cells. Journal of cellular biochemistry.

[17] Lenos K, Grawenda AM, Lodder K, Kuijjer ML, Teunisse AF, Repapi E, Grochola LF, Bartel F, Hogendoorn PC, Wuerl P, Taubert H, Cleton-Jansen AM, Bond GL (2012). Alternate splicing of the p53 inhibitor HDMX offers a superior prognostic biomarker than p53 mutation in human cancer. Cancer Res.

[18] Dewaele M, Tabaglio T, Willekens K, Bezzi M, Teo SX, Low DH, Koh CM, Rambow F, Fiers M, Rogiers A, Radaelli E, Al-Haddawi M, Tan SY (2016). Antisense oligonucleotide-mediated MDM4 exon 6 skipping impairs tumor growth. J Clin Invest.

[19] Lenos K, Jochemsen AG (2011). Functions of MDMX in the modulation of the p53-response. Journal of biomedicine & biotechnology.

[20] Liu L, Fan L, Fang C, Zou ZJ, Yang S, Zhang LN, Li JY, Xu W (2012). S-MDM4 mRNA overexpression indicates a poor prognosis and marks a potential therapeutic target in chronic lymphocytic leukemia. Cancer science.

[21] Grawenda AM, Moller EK, Lam S, Repapi E, Teunisse AF, Alnaes GI, Borresen-Dale AL, Kristensen VN, Goding CR, Jochemsen AG, Edvardsen H, Bond GL (2015). Interaction between p53 mutation and a somatic HDMX biomarker better defines metastatic potential in breast cancer. Cancer Res.

[22] Bista M, Petrovich M, Fersht AR (2013). MDMX contains an autoinhibitory sequence element. Proc Natl Acad Sci U S A.

[23] Xiong S, Pant V, Suh YA, Van Pelt CS, Wang Y, Valentin-Vega YA, Post SM, Lozano G (2010). Spontaneous tumorigenesis in mice overexpressing the p53-negative regulator Mdm4. Cancer Res.

[24] Bardot B, Bouarich-Bourimi R, Leemput J, Lejour V, Hamon A, Plancke L, Jochemsen AG, Simeonova I, Fang M, Toledo F (2015). Mice engineered for an obligatory Mdm4 exon skipping express higher levels of the Mdm4-S isoform but exhibit increased p53 activity. Oncogene.

[25] Shaw AC, Swat W, Ferrini R, Davidson L, Alt FW (1999). Activated Ras signals developmental progression of recombinase-activating gene (RAG)-deficient pro-B lymphocytes. The Journal of experimental medicine.

[26] Hulkkonen J, Vilpo L, Hurme M, Vilpo J (2002). Surface antigen expression in chronic lymphocytic leukemia: clustering analysis, interrelationships and effects of chromosomal abnormalities. Leukemia.

[27] Grier JD, Xiong S, Elizondo-Fraire AC, Parant JM, Lozano G (2006). Tissue-specific differences of p53 inhibition by Mdm2 and Mdm4. Mol Cell Biol.

[28] Bichi R, Shinton SA, Martin ES, Koval A, Calin GA, Cesari R, Russo G, Hardy RR, Croce CM (2002). Human chronic lymphocytic leukemia modeled in mouse by targeted TCL1 expression. Proc Natl Acad Sci U S A.

[29] Bezzi M, Teo SX, Muller J, Mok WC, Sahu SK, Vardy LA, Bonday ZQ, Guccione E (2013). Regulation of constitutive and alternative splicing by PRMT5 reveals a role for Mdm4 pre-mRNA in sensing defects in the spliceosomal machinery. Genes Dev.

[30] Pajares MJ, Ezponda T, Catena R, Calvo A, Pio R, Montuenga LM (2007). Alternative splicing: an emerging topic in molecular and clinical oncology. The Lancet Oncology.

[31] Venables JP, Klinck R, Koh C, Gervais-Bird J, Bramard A, Inkel L, Durand M, Couture S, Froehlich U, Lapointe E, Lucier JF, Thibault P, Rancourt C (2009). Cancer-associated regulation of alternative splicing. Nature structural & molecular biology.

[32] Wan Y, Wu CJ (2013). SF3B1 mutations in chronic lymphocytic leukemia. Blood.

